# Highly sensitive detection and quantification of the secreted bacterial benevolence factor RoxP using a capacitive biosensor: A possible early detection system for oxidative skin diseases

**DOI:** 10.1371/journal.pone.0193754

**Published:** 2018-03-01

**Authors:** Gizem Ertürk, Martin Hedström, Bo Mattiasson, Tautgirdas Ruzgas, Rolf Lood

**Affiliations:** 1 Department of Clinical Sciences Lund, Division of Infection Medicine, Biomedical Center B14, Lund University, Lund, Sweden; 2 Department of Biotechnology, Lund University, Lund, Sweden; 3 CapSenze Biosystems AB, Lund, Sweden; 4 Department of Biomedical Sciences, Faculty of Health and Society, Malmö University, Malmö, Sweden; University of Ulster, UNITED KINGDOM

## Abstract

The impact of the microbiota on our health is rapidly gaining interest. While several bacteria have been associated with disease, and others being indicated as having a probiotic effect, the individual biomolecules behind these alterations are often not known. A major problem in the study of these factors *in vivo* is their low abundance in complex environments. We recently identified the first secreted bacterial antioxidant protein, RoxP, from the skin commensal *Propionibacterium acnes*, suggesting its relevance for maintaining the redox homeostasis on the skin. In order to study the effect, and prevalence, of RoxP *in vivo*, a capacitive biosensor with a recognition surface based on molecular imprinting was used to detect RoxP on skin *in vivo*. *In vitro* analyses demonstrated the ability to detect and quantify RoxP in a concentration range of 1 x 10^−13^ M to 1 x 10^−8^ M from human skin swabs; with a limit of detection of 2.5 x 10^−19^ M in buffer systems. Further, the biosensor was highly selective, not responding to any other secreted protein from *P*. *acnes*. Thus, it was possible to demonstrate the presence, and quantity, of RoxP on human skin. Therefore, the developed biosensor is a very promising tool for the detection of RoxP from clinical samples, offering a rapid, cost-effective and sensitive means of detecting low-abundant bacterial proteins *in vivo* in complex milieus.

## Introduction

*Propionibacterium acnes* is one of the most common bacteria on human skin [1], and is involved in several skin disorders including acne vulgaris [2], and infections of prosthetic devices [3,4] due to biofilm formation on orthopedic implants [5]. Further, during microbial dysbiosis, as seen in the oxidative stress driven disease psoriasis [6], the prevalence of *P*. *acnes* is significantly decreased [7]. Due to the general high abundance of *P*. *acnes* on our skin, and its association with acne vulgaris, it has gained much scientific interest. Recently, in the omics-era, the secretome of *P*. *acnes* was published [8], demonstrating the presence of a highly secreted protein in all studied strains of *P*. *acnes* during anaerobic culturing in complex media *in vitro*. Using mass spectrometry Bek-Thomsen et al further demonstrated the presence of RoxP (e.g. PPA1939) from sebaceous follicles *in vivo*, though the abundance was lower than *in vitro* [9]. This protein, further on denoted as RoxP (Radical oxygenase of *P**ropionibacterium acnes*) was later demonstrated by us to be a unique secreted bacterial antioxidant, playing a critical role for the colonization of *P*. *acnes* on the skin, allowing the facultative anaerobe to tolerate the oxic environment [10].

The human skin is constantly being exposed to free radicals and oxidative stress, from among others UV-irradiation, activated immune cells, and regular metabolic activities [11–13]. The human body is well adapted to cope with this stress through intracellular antioxidants, like heme-oxygenase 1 that is up-regulated during UV stimulation [14]. Similarly, most bacteria, including *P*. *acnes*, have intracellular defense mechanisms against oxygen radicals, including catalases, superoxide dismutases and NADH oxidases. These antioxidants play a critical role in maintaining our health, protecting from oxidative stress and malignancies [15]. Therefore, it was suggested that the presence of the bacterial antioxidant RoxP on the skin could act as a benevolence factor, benefiting our health by protecting the skin from excess oxidative stress [10]; thus being part of maintaining our redox homeostasis. The prevalence of RoxP on skin could therefore serve as a biomarker for presence or proneness of developing oxidative diseases in individuals. However, so far no one has been able to detect and quantitate this bacterial protein from the complex skin environment.

Capacitive biosensors, a type of impedance biosensors [16], measure changes in dielectric properties, or the thickness of a dielectric layer at an electrolyte-electrode interface during the interaction between an analyte and its biorecognition element on the sensor surface [16]. Capacitive biosensors based on the use of biorecognition molecules have been used for detection of several targets, including peptides [17], toxins [18], nucleic acids [19], antibodies [20], antigens [21], and proteins [22]. To further improve on the selectivity, sensitivity, and operational stability of the biosensor surface, the use of molecularly imprinted polymers (MIPs) have been used to create biorecognition cavities on capacitive gold electrodes [23,24]. Molecular imprinting is a technique by which artificial recognition sites can be created in a polymer matrix which are complementary to the template in terms of size, shape, and chemical functionality [25,26]. MIPs are cost-effective, stable, and robust which make them superior compared to antibodies [27].

Herein we have developed a highly specific and selective capacitive biosensor for the *in vivo* detection and quantification of the skin bacterial antioxidant RoxP, using molecular imprinting. In buffer, RoxP could be detected in the attomolar range with a limit of detection of 2.5 x 10^−19^ M but could also be detected in a complex skin environment down to picomolar levels. Thus, the developed tool may be used as a highly sensitive method for detection and quantification of bacterial factors *in vivo* as exemplified by RoxP in this study.

## Materials and methods

### Materials

Acrylamide, N-hydroxymethylacrylamide (NHMA), N-isopropylacrylamide (NIPAm), N,N-methylenebisacrylamide (MBAAm), ammonium persulphate (APS), N,N,N’,N’-tetramethylethyldiamine (TEMED), Tris-HCl, Tween-20, Immunoglobulin A (IgA), Collagen, Bovine serum albumin (BSA), 3-aminopropyl-triethoxysilane (APTES), Glutaraldehyde (50%, w/v), Acryloylchloride, Triethylamine, 1-dodecanethiol, and Tyramine (99%) were all purchased from Sigma Aldrich. The capacitive biosensor and gold electrodes were supplied by CapSenze Biosystems AB, Lund, Sweden. All buffers were prepared with a Milli-Q system from Millipore. Prior to use, all buffers were filtered through a Millipore filter (pore size: 0.22 μm) and degassed for 1 h.

### Preparation of RoxP-imprinted capacitive biosensor electrodes

Glass cover slips (24 x 50 mm) which were to be used as RoxP stamps were cleaned and modified according to the protocol of Ertürk et al. [28]. Following sequential modification by treatment with 10% (v/v) APTES and 5% (v/v) glutaraldehyde [23], the glass cover slips were immersed in a 0.1 mg.mL^-1^ RoxP solution in 10 mM phosphate buffer (pH 7.4) at 4°C overnight, resulting in a RoxP immobilization on the surface of the glass cover slips.

In the second step, capacitive gold electrodes were prepared. Gold electrodes were cleaned before tyramine electropolymerization was carried out to introduce free primary amino groups on the surface as described elsewhere [21,23]. Following tyramine modification, electrodes were immersed in a solution of 30 mM acryloyl chloride and 30 mM trimethylamine in toluene overnight at room temperature to introduce vinyl groups on the surface.

In the last step, hydrogel based molecularly imprinted polymers were prepared according to the protocols described by Reddy et al [29–31]. Acrylamide (54 mg), NHMA (140 μL), and NIPAm (85.6 mg) were used as functional monomers and methylenebisacrylamide (MBAAm) (9.5 mg) was used as a cross-linker [30]. Functional monomers and cross-linker were dissolved in 820 μL of Milli-Q water. TEMED (20 μL, 5% (v/v)) was added to the polymer matrix and purged with nitrogen gas for 5 min. Freshly prepared APS (20 μL, 10% (w/v)) was added to the mixture, and 1.5 μL of this solution was pipetted onto the modified gold electrode surface before the RoxP stamp was brought into contact with the monomer treated electrode. Polymerization continued for 5 h at room temperature after which the RoxP stamp was gently removed from the surface, and the electrode was rinsed with 10 mM phosphate (pH 7.4) buffer. In order to cover pinholes in the insulating layer on the gold surface, electrodes were immersed in 1-dodecanethiol (10 mM in ethanol) for 20 min. After rinsing the electrodes with water and drying them with nitrogen, a RoxP-MIP electrode was inserted into the electrochemical flow cell of the capacitive system for analysis (CapSenze HB, patent no. US9304096 B2, 2016).

**Fig 1:**
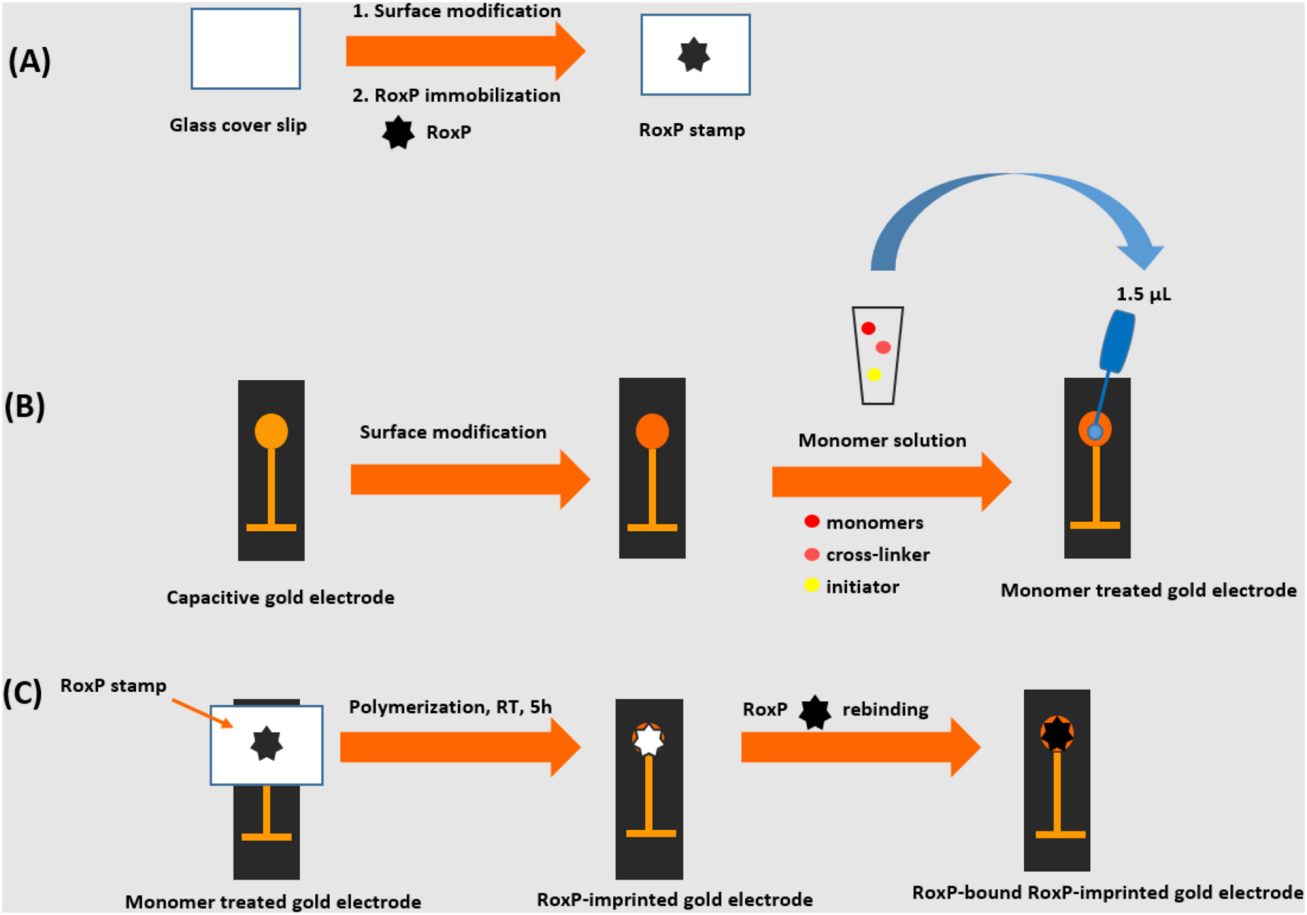
Schematic representation of the imprinting of RoxP onto the capacitive biosensor. (A) Preparation of the glass cover slips (protein stamps), (B) surface modifications of the capacitive gold electrodes and treatment with the monomer solution, and (C) imprinting of RoxP onto the gold electrode surface and rebinding of RoxP to the imprinted cavities.

A schematic presentation of the preparation of RoxP imprinted biosensor is shown in Fig 1. A non-imprinted (NIP) electrode was also prepared as a control in the presence of exactly the same monomer composition but in the absence of RoxP. A glass slide which was not containing any protein on the surface was used as the stamp for the preparation of the NIP electrode.

### Surface characterization of RoxP-MIP capacitive electrodes with scanning electron microscopy (SEM)

For SEM, specimens were mounted on aluminum holders with adhesive carbon tape and sputtered with palladium/gold to create a 10 nm thick layer. The samples were examined in a DELPHI correlative light and electron microscope (Phenom-World) at the IQ Biotechnology Platform, Lund University.

### Real time capacitive measurements with the RoxP-MIP capacitive system

Capacitance measurements were performed based on the current pulse method introduced by Erlandsson et al [20]. The capacitance measurement was performed by using a current pulse method which is based on the principle of an electrical double layer and the electrode-solution interface. This current pulse technique offers some advantages over the commonly used potential pulse technique. The use of microampere constant current helps the electrodes to be more resistant against external electronic disturbances which can cause to inaccurate measurement and poor baseline stability. The calculation of capacitance is done in a way that the potential response is linear to the pulse of current which makes it simple and more accurate. In this system, four values of capacitance are calculated from one cycle of current supply. One analysis cycle was started with the injection of regeneration buffer (25 mM glycine-HCl, pH 2.5, including 0.05 M Tween-20) to regenerate the surface, followed by equilibration in running buffer (50 mM Tris, pH 7.4) for 25 min in total before introduction of the sample to be analyzed. RoxP (1.0 x 10^−18^ M—1.0 x 10^−13^ M) dissolved in running buffer was injected sequentially into the system. All samples were measured in triplicates. After injecting the samples into the capacitive system, an average of the last five readings was calculated automatically by the CapSenze software and the calibration graphs were obtained by plotting the change in capacitance (-pF.cm^-2^) vs the log concentration of the analyte. The binding of RoxP to the surface resulted in a decrease in the total capacitance of the system according to the equation:

$$\frac{1}{C(tot)}\;=\;\frac{1}{C(ins)}+\frac{1}{C(bio)}+\frac{1}{C(dl)}$$

where total capacitance is equal to the capacitance contributions of insulating, biosensing and diffuse layers, respectively.

### Selectivity of the RoxP-MIP biosensor

Selectivity experiments were carried out in order to show the selectivity of the system for RoxP versus other competing proteins. For selectivity, IgA, collagen, and BSA were used as competing proteins; and a NIP electrode as a negative control. Further, culture media from a Δ*roxP P*. *acnes* strain [32] was compared to wildtype culture media. The changes in capacitance (ΔC) after the injection of competing proteins were compared with the change due to RoxP injection, as well as the responses on RoxP-MIP and NIP electrodes. The measured values were used to calculate selectivity (k) and relative selectivity coefficients (k’). The selectivity coefficients (k) were calculated as ΔC_RoxP_/ΔC_competitor_. Relative selectivity coefficient was calculated as k_RoxP-MIP_/k_NIP_ for IgA, collagen and BSA. All measurements were performed in triplicates.

### Collection of skin swab samples

A skin surface area (cheek, forehead) of 4x4 cm was gently rubbed with a cotton swab (ESwab, Copan Diagnostics) for 20 seconds on one middle-aged (30–35 years) man and woman without any current skin disorders. The swab was put back into its container, gently shaken, and left at 4°C. Cells and debris were collected by centrifugation (4000 g, 15 min), and the supernatant frozen until analysis on the capacitive biosensor. All samples were collected with an informed written consensus of the individuals, according to the Declaration of Helsinki. An ethical approval to collect the samples was granted by the Lund University ethical committee (Dnr 2016/465).

### Detection of RoxP on skin

A calibration graph of RoxP in Copan ESwab storage solution was performed in the concentration range of 1.0 x 10^−13^–1.0 x 10^−9^ M to take into account the background signal from the media itself. Extracted skin samples were diluted 1/10–1/10^5^ in 50 mM Tris-HCl (pH 7.4), and injected into the biosensor system. All measurements were performed in triplicates.

## Results

### Surface characterization of bare and RoxP-MIP gold electrodes

**Fig 2:**
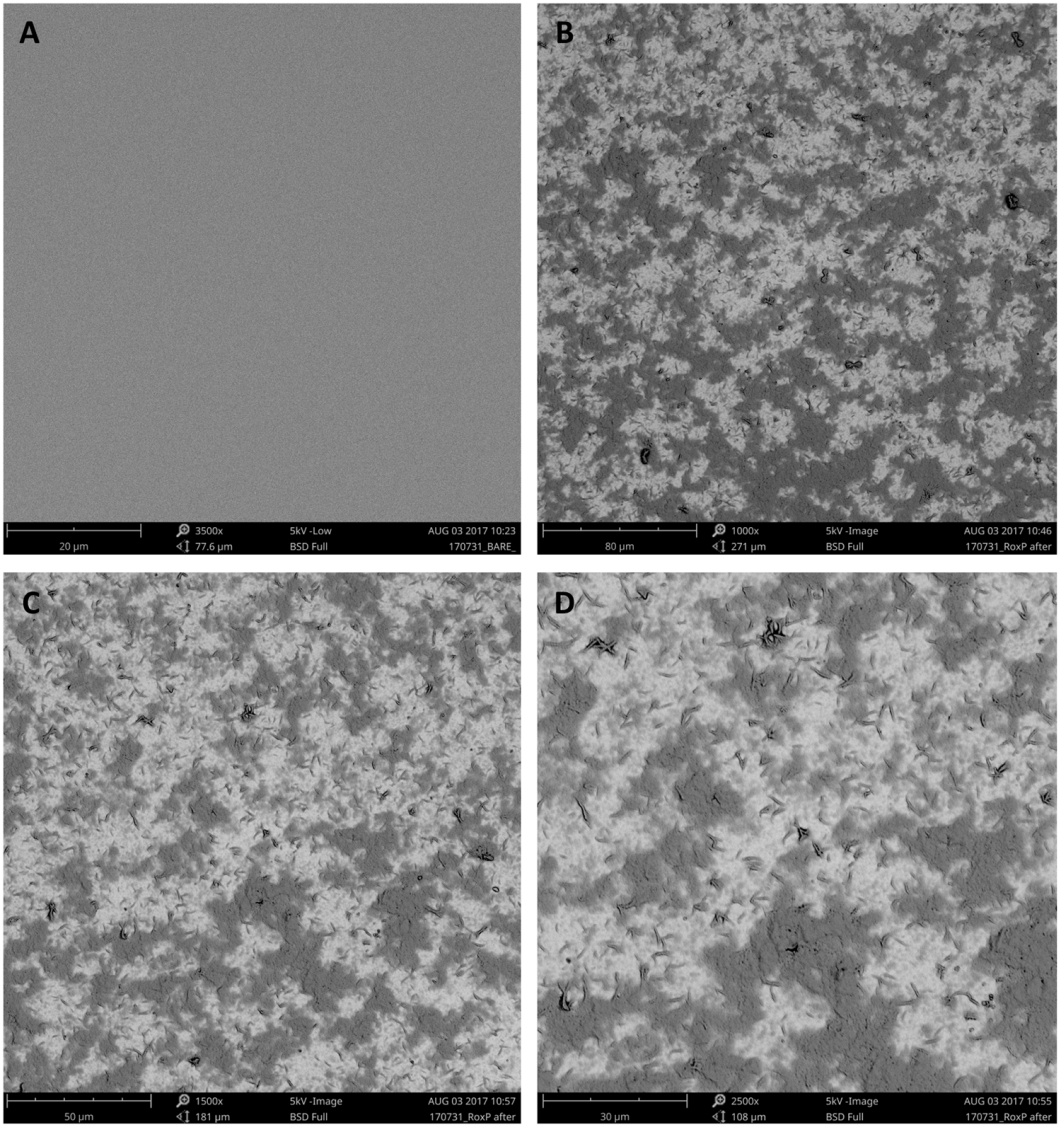
SEM micrographs of RoxP imprinted capacitive gold electrodes. (A) Bare gold electrode, and (B-D) RoxP-imprinted electrodes in different magnifications (B: 1,000x; C: 1,500x; D: 2,500x).

In order to be able to detect and quantify the bacterial benevolence factor RoxP, a gold electrode was covered with a polymer matrix having cavities based on RoxP. In comparison to a bare gold electrode (Fig 2A), the RoxP imprints on the RoxP-MIP electrode could be visualized as small non-distinctive cavities by SEM (Fig 2B–2D).

### Real-time detection of RoxP with RoxP-MIP capacitive biosensors

**Fig 3:**
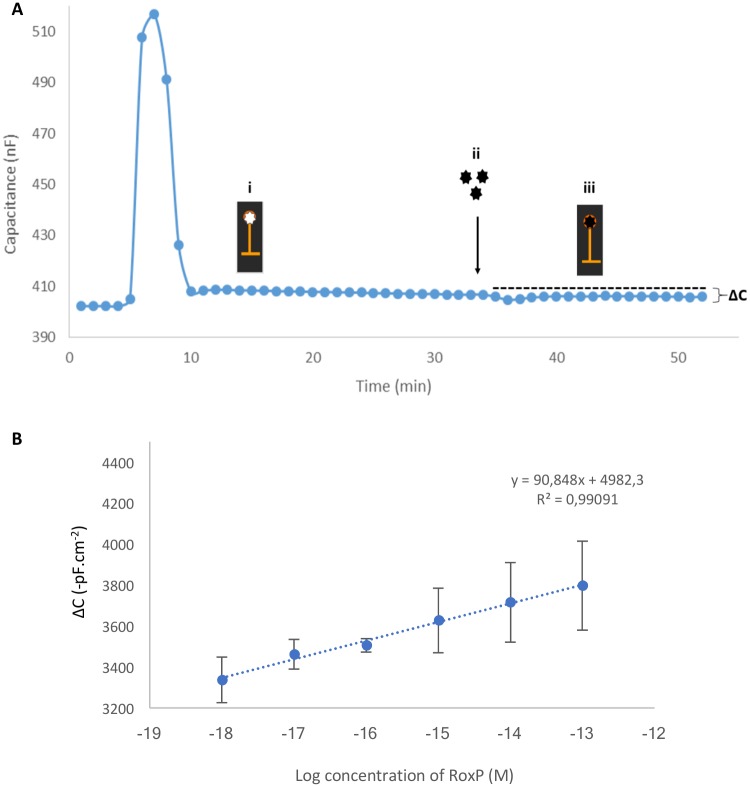
Binding of RoxP to a MIP-electrode. (A) Actual sensorgram showing real-time binding of RoxP to the RoxP-imprinted gold electrode indicating i) a stable baseline with the RoxP-electrode having template-specific cavities (white star), ii) injection of sample, and iii) binding of RoxP to the electrode, resulting in a change in capacitance. (B) Using different concentrations of RoxP, a calibration curve was established and fitted to a linear regression model. A running buffer of 50 mM Tris pH 7.4, and a regeneration buffer of 25 mM glycine-HCl pH 2.5 supplemented with 0.05 M Tween-20 was used, operating the instrument at a speed of 100 μl/min and a sample volume of 250 μl.

To verify that the molecular imprints of RoxP in the polymer could interact with free RoxP, a dilution series of RoxP (1.0 x 10^−18^–1.0 x 10^−13^ M) was sequentially injected into the capacitive biosensor flow system. The ΔC (change in capacitance; Fig 3A) increased linearly with increasing concentrations of RoxP with a good fitting to a linear regression model (R^2^ = 0.99) (Fig 3B). The limit of detection was calculated as 2.5 x 10^−19^ M based on IUPAC guidelines [33].

### The RoxP-MIP capacitive biosensor is highly selective for RoxP

**Fig 4:**
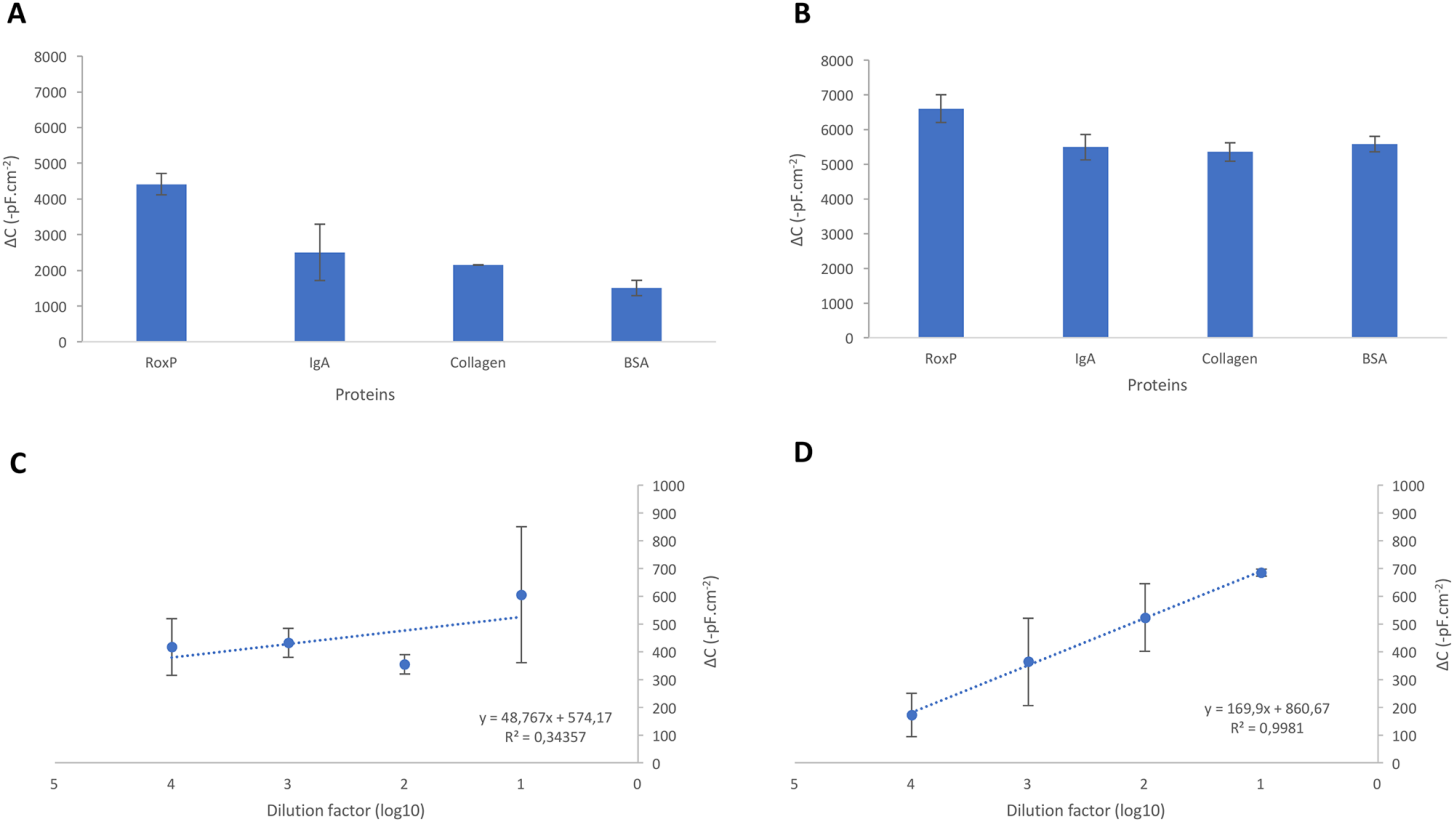
The RoxP-MIP is highly selective. RoxP (10^−8^ mg/mL for MIP, 10^−3^ for NIP) or competitor protein (10^−3^ mg/mL) were added to either a RoxP-MIP electrode (A) or to the non-selective NIP electrode (B). A dilution series of culture media from either a wildtype *P*. *acnes* (C; RoxP^+^) or its isogenic *roxP* mutant (D; RoxP^-^) were analyzed on the RoxP-MIP electrode. A running buffer of 50 mM Tris pH 7.4, and a regeneration buffer of 25 mM glycine-HCl pH 2.5 supplemented with 0.05 M Tween-20 was used, operating the instrument at a speed of 100 μl/min and a sample volume of 250 μl.

When analyzing environmental or patient samples, the complexity of the material is often contributing to either blocking the signal or creating false positives; mainly due to the low abundance of the analyte and high abundance of interfering material. Therefore, in order to detect low-abundant molecules in the presence of high-abundant molecules, the biosensor must have high selectivity and specificity. Due to the presence of RoxP on skin, we challenged the system with two other common skin proteins IgA and collagen. Further, BSA was included as a general control protein for selectivity purposes. Despite a concentration difference of approximately 10^4^, favoring the competing proteins (IgA: 6.2 x 10^−9^ M; collagen: 3.7 x 10^−9^ M; BSA: 1.45 x 10^−8^ M), RoxP still generated higher response levels (Fig 4A). No difference in affinity between proteins could be observed using a NIP electrode (Fig 4B). Based on the data the selective coefficient (k) as well as the relative selectivity coefficient (k’) was calculated (Table 1), indicating the preference of RoxP-MIP to interact with RoxP (k_MIP_ 3.09–5.14) while non-specific electrodes (NIP) did not show any preference for RoxP interaction (k_NIP_ 1.02–1.06).

**Table 1:** Selectivity coefficients of RoxP-MIP capacitive biosensor.

Protein	Concentration (mg/mL)	ΔMIP	ΔNIP	k_MIP_	k_NIP_	k’
RoxP	10^−8^	7744	5696	-	-	-
Collagen	10^−3^	2152	5491	3.60	1.04	3.47
IgA	10^−3^	2505	5352	3.09	1.06	2.90
BSA	10^−3^	1507	5580	5.14	1.02	5.03

ΔMIP: Capacitance change of the MIP electrode for the proteins; ΔNIP: Capacitance change of the NIP electrode for the proteins; k_MIP_: selectivity coefficient of the MIP electrode versus competing proteins; k_NIP_: selectivity coefficient of the NIP electrode versus competing proteins; k’: relative selectivity coefficient of the MIP electrode versus the NIP electrode.

To further verify the selectivity of the RoxP-MIP, culture media from a wildtype *P*. *acnes* and its isogenic *roxP* mutant was analyzed on the capacitive biosensor. The wildtype *P*. *acnes* displayed a well-fitted linear regression (R^2^ = 0.998) with a high k-value, indicating an efficient response to RoxP (Fig 4D). However, the isogenic *roxP* mutant, lacking RoxP in the culture media, did not display a linear increase between the dilutions (R^2^ = 0.344), but rather a horizontal base line (Fig 4C).

### RoxP can be detected and quantified from skin swabs

**Fig 5:**
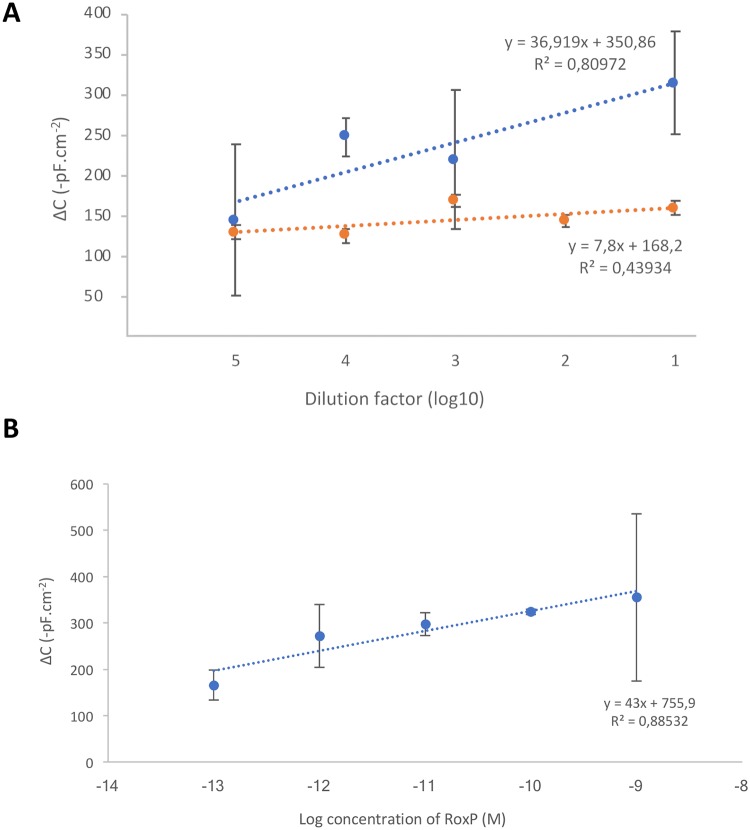
RoxP detection on skin *in vivo*. (A) RoxP detection with the RoxP-MIP capacitive biosensor for a RoxP spiked sample containing the same buffer composition as skin swabs, (B) or from non-spiked samples (control sample; orange) or skin swabs (blue) using a dilution ratio of 1/10. A running buffer of 50 mM Tris pH 7.4, and a regeneration buffer of 25 mM glycine-HCl pH 2.5 supplemented with 0.05 M Tween-20 was used, operating the instrument at a speed of 100 μl/min and a sample volume of 250 μl.

Since RoxP can be found on skin, it is imperative to investigate the ability of the capacitive biosensor to detect and quantify RoxP in such a complex sample. A mock skin swab was spiked with a dilution series of RoxP to generate a calibration curve in the specific media (Fig 5A). As a proof of principle that the biosensor can detect RoxP from skin *in vivo*, a swab from the forehead of a healthy middle-aged man was measured in a dilution series to verify the dose-dependency, confirming that the signal was not due to binding of interfering material (Fig 5B). Extrapolating the change in capacitance to a RoxP concentration demonstrated the presence of 10^−9^ M (9.4 nM ± 15.9 nM) RoxP in the skin swab sample, equivalent to 8.25 ng RoxP/cm^2^ skin (± 15.5 ng). The presence of other components in the sample matrix sometimes prevents the accurate and very sensitive measurement of the target. This can be one of the reasons why the LOD value obtained from skin swab is higher compared to the LOD value obtained from the buffer. Further studies will be able to investigate gender, age, and disease specific abundancies of RoxP on skin using this novel developed highly sensitive, specific, and selective biosensor.

## Discussion

The impact of bacteria in both health and disease has been the focus for many researchers, and several factors contributing to disease (e.g. virulence factors) have been characterized. As is often the case with scientific questions, we need to simplify the experimental settings in order to examine the function of the target protein/factor. The beauty of the simplicity is the more conclusive answers it generates; the drawback being the loss of the thousands of natural factors also affecting the system. In many instances, the lack of human *in vivo* data can be attributed to the difficulty of detecting and quantitating the analyte. Factors to be studied in blood, saliva, skin, tissues, or other complex samples often need a high degree of processing before any analysis can take place, with high-abundant proteins/molecules often negatively affecting the readout [34]. Here we developed a highly sensitive, selective, and specific biosensor based on molecular imprinting of an antioxidant (RoxP) from the skin commensal *P*. *acnes*, able to detect and quantitate attomolar quantities of RoxP under optimized conditions, Importantly, we were able to detect RoxP *in vivo* on skin.

The biological function(s) of RoxP is still under characterization. We recently showed the importance of RoxP for colonization of *P*. *acnes* on skin, as well as survival in oxic environments *in vitro* [10]. However, the actual oxic conditions in the pilosebaceous follicles, the main habitat of *P*. *acnes*, and the necessity of RoxP to colonize this region of the skin, has not been investigated. The lowered abundance of RoxP in the sebaceous follicles compared to *in vitro* suggests that RoxP may partake in spreading of *P*. *acnes* (e.g. exposure to oxygen) rather than actual colonization in the sebaceous follicles. Due to its high affinity to heme, it has also been argued that the secreted porphyrins of *P*. *acnes* [35] may be the actual substrate of RoxP [10], reducing inflammatory UV-oxidized porphyrins. Further molecular investigations within this area will be needed to shed light upon this interaction.

In order to achieve the high sensitivity, several parameters have to be taken into account, the running buffer being but one of them. Earlier studies by El-Sharif et al, using a similar monomer composition as in our study, demonstrated the high selectivity of their system in Tris-HCl, while PBS reduced the selectivity [30]. In slightly basic conditions, Tris buffer will present three non-dissociated hydroxyl groups that can form hydrogen bonds with the MIP and the template protein, providing ideal conditions for a native protein interaction with the cavities within the MIP. While the buffer conditions may partly influence the selectivity, the monomer composition of the MIP is critical. The usage of hydrogel based molecular imprinted polymers (Hydro-MIPs) have shown high selectivity for template proteins as compared to NIP polymers [29,31,36]. Thus, even though highly selective, an even higher selectivity can likely be achieved for RoxP by optimizing the ratio of the monomers used for the polymer.

The issue of high background is prevalent in many methods. Due to the high sensitivity of the MIP biosensor, and thus the ability to dilute the sample several 10-folds, the problem with the complexity of the sample is reduced. Still, components of the sample may interact with the MIP, as seen with the culture media from the isogenic *roxP* mutant *P*. *acnes* strain. This interaction, likely due to short peptides present in the broth, is however of a much lower affinity than that of the analyte-MIP interaction, as evident by the significant fit of a linear regression model to a spiked culture media sample.

In this study we have focused on the secreted bacterial protein RoxP, an antioxidant that has been speculated to partake in the protection and redox homeostasis of the skin [10]. Since the skin is constantly exposed to oxidative stress through UV-irradiation, activated immune cells, and regular metabolic processes [6], the presence of an efficient antioxidant on the skin may prove beneficial. The involvement of *P*. *acnes* in several diseases has so far mainly been indicative rather than conclusive, much owing to its high prevalence on the skin. However, it is commonly reported to be underrepresented in skin dysbiosis (e.g. psoriasis) [7], diseases with a known element of oxidative stress [6]. Therefore, the ability to measure the presence of *P*. *acnes*, and specific proteins (RoxP) in real-time *in vivo* would generate valuable data allowing us to further evaluate the importance of this skin commensal and its role in health and disease. Earlier investigations have demonstrated the presence of RoxP *in vitro* and *in vivo* [8,9], but none of the methods employed (*e*.*g*. SDS-PAGE and MS/MS) are suitable for absolute quantification, nor relative quantification, due to the differences in molecular mass and ability of peptides to fly in the mass spectrometer.

We took a first step towards enabling absolute quantification of RoxP by developing a RoxP-MIP biosensor, optimizing it for detection and quantification of RoxP in the complex skin environment, detecting low ng quantities on the skin; a concentration comparing well to that of extrapolated *in vitro* measurements. For comparison, though the skin hardly can be described as a flat organ, a monolayer of proteins on a flat surface will reach quantities close to 300 ng/cm^2^ indicating that RoxP is highly abundant in the skin of healthy individuals. It should however be noted that we only took a skin swab, and that skin samples from different compartments (e.g. sebaceous follicles, scrub-wash fractions, etc) would generate different numbers; in particular since it is still unknown whether it is only the surface associated *P*. *acnes* expressing RoxP, or if also the follicular *P*. *acnes* express this antioxidant. Further research, taking advantage of this highly sensitive and selective biosensor, will shed light upon the *in vivo* role of RoxP in skin health and disease.

## Conclusion

Herein, we have developed a highly sensitive, selective, and specific biosensor for the detection and in real-time quantification of the bacterial benevolence factor RoxP. Not only can this method be used as a research tool, or diagnostics to measure the presence of RoxP on skin. Rather, this method can be generalized to target any protein/biomolecule of interest within a complex milieu for a highly sensitive and selective detection and quantification, as exemplified with the quantification of the putative oxidative stress biomarker RoxP on the skin *in vivo*
